# Genetic Associations of Interleukin-related Genes with Graves’ Ophthalmopathy: a Systematic Review and Meta-analysis

**DOI:** 10.1038/srep16672

**Published:** 2015-11-18

**Authors:** Kah Hie Wong, Shi Song Rong, Kelvin K. L. Chong, Alvin L. Young, Chi Pui Pang, Li Jia Chen

**Affiliations:** 1 Department of Ophthalmology and Visual Sciences, The Chinese University of Hong Kong, Hong Kong, China; 2 Department of Ophthalmology and Visual Sciences, Prince of Wales Hospital, Hong Kong, China

## Abstract

Graves’ ophthalmopathy (GO) is the commonest extra-thyroidal manifestation of Graves’ disease (GD). Associations between interleukin-related (IL) gene polymorphisms and GO have been reported in different populations. We aim to confirm such associations by conducting a meta-analysis. Totally 382 publications were retrieved in MEDLINE and EMBASE up to 25/2/2015. After removing the duplicates and assessing the studies, we retrieved 16 studies that met the selection criteria for meta-analysis, involving 12 polymorphisms in 8 IL-related genes, and 1650 GO cases and 2909 GD controls. The summary odds ratio (OR) and 95% confidence intervals (CI) were estimated. We found one polymorphism in *IL1A* (rs1800587, c.-889C>T) showing a suggestive association with GO in the meta-analysis (allelic model [T vs. C]: OR = 1.62, 95% CI: 1.00–2.62, P = 0.050, *I*^*2*^ = 53.7%; recessive model [TT vs. TC + CC]: OR = 2.39, 95% CI: 1.07–5.37, P = 0.039, *I*^*2*^ = 23.6%; heterozygous model [TC vs. CC]: OR = 1.52, 95% CI: 1.04–2.22, P = 0.034, *I*^*2*^ = 37.0%). No association with GO was detected for the other 7 genes (*IL1B*, *IL1RA*, *IL4*, *IL6*, *IL12B*, *IL13* and *IL23R*). Our results thus indicate that *IL1A* is likely to be a genetic biomarker for GO. Further studies with larger sample sizes are warranted to confirm the associations of *IL1A* and other IL-related genes with GO.

Graves’ ophthalmopathy (GO), also known as thyroid-associated orbitopathy (TAO), is the commonest extra-thyroidal manifestation of Graves’ disease (GD), present in reportedly 25–50% of cases with GD^1^,^2^. It is also the commonest adult orbital disorder worldwide^3^. GO is characterized by lid retraction, lid lag, swelling and erythema of conjunctiva and periocular tissues, restrictive strabismus and proptosis^4^. Around 3–5% of GO patients develop sight-threatening complications, such as globe subluxation, corneal ulceration due to exposure keratopathy and optic neuropathy, which may result in irreversible visual impairment or even blindness if not treated promptly and properly^3^.

GO is a complex disease with interactive genetic and environmental factors^5^. Although the pathological mechanisms of GO are not completely understood^6^, cytokines, especially interleukins (ILs), are evidently involved^4^. Enlarged extraocular muscles and expansion of orbital adipose tissues^7^ were shown histologically with infiltration of activated T cells, B cells and macrophages^8^,^9^,^10^. Interleukins IL1RA^11^, IL1B^12^, IL4^12^, IL6^12^ and IL10^12^ were detected in the affected tissues of GO patients. In GO patients, higher levels of ILs were found in orbital tissues (IL1B^13^, IL7^14^, IL8^13^ and IL10^13^), tears (IL7^14^) and serum (IL1RA^11^, IL6^15^,^16^ and sIL-6R^16^). In addition, the expressions of IL1B and IL6 mRNA in the orbital adipose tissues were positively correlated with the radiological orbital volume in GO patients^12^. It has been speculated that Th1 lymphocytes and associated Th1-like cytokines (IL1B, IL2, IL12, INFG and TNFA) predominate in^17^, and promote the inflammatory, active phase of GO, while the Th2 family of cytokines (IL4, IL5 and IL10)^18^ affect the later fibrotic, inactive phases of the disease^19^,^20^.

Emerging studies have shown positive associations of polymorphisms in the IL-related genes (including ILs, interleukin receptors and receptor antagonists) with GO. Over 50 genetic polymorphisms of 17 IL-related genes, namely *IL1A*^21^,^22^, *IL1B*^21^,^22^,^23^,^24^, *IL1R*^21^, *IL1RA*^21^,^25^,^26^,^27^, *IL2*^28^, *IL3*^29^, *IL4*^29^,^30^,^31^, *IL5*^29^, *IL6*^28^,^32^, *IL8*^33^, *IL9*^29^, *IL10*^31^, *IL12B*^28^,^34^,^35^, *IL13*^29^,^30^,^36^,^37^,^38^, *IL18*^39^, *IL21*^40^ and *IL23R*^41^,^42^,^43^, were reported in GO among different populations. However, the associations of these polymorphisms were inconsistent across different studies. For example, a single-nucleotide polymorphism (SNP), rs16944, in the *IL1B* gene was significantly associated with GO in a Chinese cohort from mainland China^22^, but not in Taiwan Chinese^24^, Caucasians^23^ or Iranian^21^. Also, the *IL23R* SNPs rs2201841 and rs10889677 were associated with GO in Caucasians^41^ but not in Japanese^42^,^43^. Therefore, we conducted a systematic review and meta-analysis to summarize the associations of reported IL-related genes with GO.

## Results

### Characteristics of identified studies

**Figure 1:**
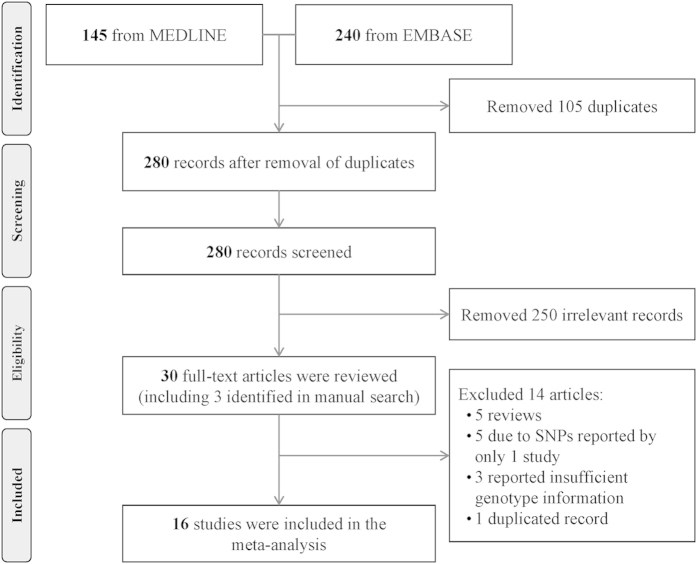
Flow diagram of study selection process.

In the literature search, a total of 385 records, published between May 1, 1989 and February 25, 2015 were retrieved from the EMBASE and MEDLINE database. Among them, 105 were duplicated records. From the remaining 280 articles, we found 27 to be relevant according to our study criteria^1^,^21^,^22^,^23^,^24^,^25^,^26^,^27^,^28^,^29^,^31^,^32^,^33^,^34^,^35^,^38^,^39^,^40^,^41^,^42^,^43^,^44^,^45^,^46^,^47^,^48^,^49^. We further manually screened the reference lists and identified another 3 relevant studies^30^,^36^,^37^. Therefore, 30 articles were studied. We excluded 5 reviews^1^,^44^,^47^,^48^,^49^, 3 studies in which there were no sufficient genotypic or allelic data after communication with the authors^38^,^45^,^46^, and 1 study with duplicated samples^43^. Also excluded were 5 other studies, in which the SNPs were reported only once in the literature and not eligible for meta-analysis^27^,^33^,^35^,^39^,^40^. Finally, 16 studies investigating totally 12 genetic variations in 8 IL-related genes were included into this meta-analysis (Fig. 1).

The 16 studies involved a total of 1,650 GO patients and 2,909 GD controls (GD without GO) recruited from Caucasian^23^,^25^,^26^,^32^,^36^,^41^, Chinese^22^,^24^,^29^,^30^, Japanese^34^,^37^,^42^ and Iranian^21^,^28^,^31^ populations. The sample sizes of GO patients ranged from 44^26^ to 200^24^, and GD controls from 28^25^ to 569^22^. The diagnostic criteria for GD were stated in all included studies except one^23^. In 12 studies, GO was classified according to the NOSPECS criteria (Supplementary Table 1) and only GO with NOSPECS Class 2 (or 3) and above were included^21^,^22^,^23^,^24^,^26^,^28^,^29^,^31^,^32^,^34^,^36^,^37^. The other 4 studies did not report the definition of GO^25^,^30^,^41^,^42^ (Table 1). Moreover, 9 studies reported the results of tests for Hardy-Weinberg equilibrium (HWE) in controls^21^,^22^,^23^,^28^,^29^,^30^,^31^,^34^,^37^, and there are 5 studies testing both genetic and non-genetic risk factors (e.g. gender, family history and smoking)^21^,^24^,^30^,^32^,^37^.

### Genetic associations of IL-related genes with GO

**Figure 2:**
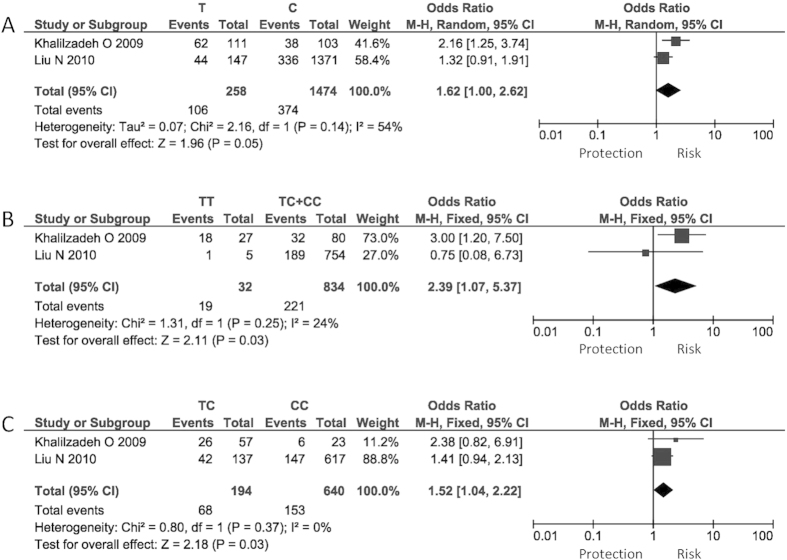
Association of *IL1A* rs1800587 with GO allelic, recessive and heterozygous models. (**A**) Allelic model; (**B**) Recessive model; (**C**) Heterozygous model.

We meta-analyzed 12 variations in 8 IL-related genes, including *IL1A* (rs1800587), *IL1B* (rs1143634 and rs16944), *IL1RA* (A2, two copies of an 86-bp tandem repeat in intron2), *IL4* (rs2070874), *IL6* (rs1800795), *IL12B* (rs3212227), *IL13* (rs1800925 and c.-2044G>A) and *IL23R* (rs10889677, rs2201841 and rs7530511). The number of studies on each variation ranged from 2 to 4. Only 1 SNP, *IL1A* rs1800587, showed a marginally significant association with GO in allelic (T vs. C, summary odds ratio [OR] = 1.62, P = 0.050, I^2^ = 53.7%), recessive (TT vs. TC + CC, OR = 2.39, P = 0.039, I^2^ = 23.6%) and heterozygous (TC vs. CC, OR = 1.52, P = 0.034, I^2^ = 0) models. This SNP was reported in 2 studies, involving a total of 240 GO patients and 626 GD controls (Table 2; Fig. 2). However, the associations were not significant after Bonferroni correction (P > 0.01).

The other 11 variations in 7 genes did not show a significant association with GO in any inheritance models (P > 0.05; Table 2). Among the insignificant polymorphisms, *IL1B* rs1143634, *IL1RA* A2/non-A2 and *IL23R* rs7530511 showed no heterogeneity among studies (I^2^ = 0), whilst *IL1B* rs16944 (I^2^ ≤ 46.2%), *IL4* rs2070874 (I^2^ ≤ 41.3%), *IL6* rs1800795 (I^2^ ≤ 80.9%), *IL12B* rs3212227 (I^2^ ≤ 85.4%), *IL13* rs1800925 (I^2^ ≤ 31.5%) and c.-2044G>A (I^2^ ≤ 46.4%), and *IL23R* rs10889677 (I^2^ ≤ 89.7%) and rs2201841 (I^2^ ≤ 86.3%) showed moderate to high heterogeneities (Table 2).

To explain the heterogeneity, we performed subgroup analysis by ethnicity. Due to the limited number of studies, we only tested the associations of 2 SNPs (*IL1B* rs16944 and *IL4* rs2070874) in Chinese. However, these 2 SNPs did not show significant association with GO (P > 0.05), with low to moderate heterogeneities (Supplementary Table 2).

### Assessment of potential biases and sensitivity analysis

Lacka *et al.* compared a subgroup of patients with GD associated with GO from the onset and a subgroup contained patients in whom GO developed from 6 months to 7 years from the onset of GD^23^. To avoid selection bias, we conducted sensitivity analysis by excluding this study and keeping only patients with GD without GO as controls from the meta-analysis of *IL1B* rs16944 and rs1143634. The associations remained insignificant (Supplementary Table 3). In the quality assessment of studies using the Newcastle Ottawa Scale (NOS), all of the studies were assigned 7 or more stars, indicating low risk of introducing biases. Therefore, no study was excluded from the meta-analysis due to poor quality (Supplementary Table 4). Moreover, for SNPs reported in 3 or more studies (i.e., *IL1B* rs1143634 and rs16944, *IL4* rs2070874 and *IL13* rs1800925), sensitivity analyses were performed by sequentially omitting one study at a time. The insignificant associations remained unchanged (P > 0.05; data not shown). There was no significant publication bias detected by the funnel plots (data not shown) and Egger’s test (Table 2 and Supplementary Table 3).

## Discussion

This study has, for the first time, summarized the associations of IL-related genes with GO. Among the 11 reported genes, we performed meta-analyses on 12 polymorphisms in 8 genes. Unexpectedly, we found only one SNP, *IL1A* rs1800587 (c.-889C>T), being marginally associated with GO. No significant association was detected for SNPs in the other 7 genes (*IL1B*, *IL1RA*, *IL4*, *IL6*, *IL12B*, *IL13* and *IL23R*), among which *IL1B* rs1143634, *IL1RA* A2/non-A2 and *IL23R* rs7530511 showed no heterogeneity across the study populations.

The *IL1A* SNP rs1800587 showed a suggestive association with no to moderate heterogeneities in different genetic models. This SNP was reported in 2 studies^21^,^22^. Although a significant association was reported only in one study^21^, the effect of the risk allele T pointed to the same direction in the both studies (OR = 2.16^21^ and OR = 1.32^22^). The heterogeneity could be due to the relatively small sample size in the study of Khalilzadeh *et al.* (about 50 cases and 50 controls), the ethnic differences in linkage disequilibrium structures, and the differences in the minor allele frequencies (MAF) of rs1800587 (T) between Iranian (about 43.0%)^21^ and Chinese (about 10%)^22^. Notably, however, since the P values did not survive the Bonferroni correction for multiple testing, the genetic association of the *IL1A* SNP with GO has yet to be confirmed in further studies with larger sample sizes.

IL1A, a major member of the IL1 superfamily, is the prototype pro-inflammatory and a potent pleiotropic cytokine involved in acute or chronic inflammation^50^. Associations between IL1A and GO were demonstrated in biochemical, histological, immunological and genetics studies. There were significant differences in the serum IL1A levels between controls and GO patients, and for the latter, before and after corticosteroid, corticosteroid with orbital irradiation, or decompression^51^. IL1A immunoreactivity was detected in the orbital tissues, their fibroblast cultures and supernatants from 5 out of 6 GO patients, but absent in those derived from 5 normal individuals^52^. An *in vitro* study demonstrated the induction of intercellular adhesion molecule 1 (ICAM-1), endothelial leukocyte adhesion molecule 1 (ELAM-1) and vascular cell adhesion molecule 1 (VCAM-1), which promote T cell chemotaxis upon the exposure of endothelial cells generated from retrobulbar tissues to IL1A^53^. The proliferation of orbital fibroblasts from GO patients was stimulated by IL1A, which has no effect on normal orbital fibroblasts^54^. Transcription of prostaglandin endoperoxidase H synthase-2 (an inflammatory cyclooxygenase that produces prostaglandin E2 and contributes to orbital inflammation in GO^55^) in orbital fibroblasts by leukoregulin (a product of activated T lymphocytes) was found to be mediated through an intermediate induction of IL1A^56^. In our meta-analysis, we found the *IL1A* SNP rs1800587 as a potential susceptibility genetic marker for GO, confirming the involvement of IL1A in the disease. In fact, the *IL1A* SNP rs1800587, located in the 5’ untranslated region (c.-889C>T), had been associated with autoimmune diseases including ankylosing spondylitis^57^, systemic lupus erythematosus^58^, psoriatic arthritis^59^ and Behcet’s disease^60^. *IL1A* and its SNP rs1800587 could thus play a role in the pathogenesis of autoimmune diseases including GO.

Except for *IL1A*, SNPs in other reported genes did not show a significant association with GO in our meta-analysis. Three SNPs showed no association with GO in all of the tested populations (P > 0.05) with no heterogeneity, including *IL1B* rs1143634 (Caucasians, Iranian and Chinese), *IL1RA* A2/non-A2 (Caucasians) and *IL23R* rs7530511 (Caucasians and Japanese). They are not likely to be genetic markers for GO. Another 6 insignificant SNPs (*IL1B* rs16944, *IL4* rs2070874, *IL6* rs1800795, *IL13* rs1800925 and c.-2044G>A, and *IL23R* rs10889677) also lacked significant association in any of the studies with mild to high heterogeneities. In contrast, *IL12B* rs3212227^28^ and *IL23R* rs2201841^41^ showed significant associations with GO in Iranian and Caucasians, respectively, but not in Japanese^34^,^42^. In our meta-analysis, no significant association was found for these 2 SNPs by using the random-effect model, with moderate to high heterogeneities. Of note, the *IL23R* SNP rs2201841 was also significantly associated with other autoimmune diseases, such as Crohn’s Disease^61^,^62^ and rheumatoid arthritis^62^. Therefore, further replication of these 2 SNPs, *IL12B* rs3212227 and *IL23R* rs2201841, in GO among different populations are warranted.

Two *IL1B* SNPs, rs1143634 and rs16944, showed no association with GO. The summary results of the *IL1B* gene in our meta-analysis were inconsistent with that in the study of Liu *et al.*^22^. In this study, we used patients with GD but without GO as controls, with a view to assess the effects of the gene SNPs on GO in a background of GD. In contrast, Liu’s group compared GO patients with healthy subjects and detected a significant association^22^. Thus, our results cannot be compared directly with that of Liu *et al.* Further studies are needed to confirm if the *IL1B* SNPs are genuine markers differentiating GO patients from normal subjects.

IL1RA acts as a competitive inhibitor of IL1A and IL1B and blocks IL1-mediated cellular activities^63^, such as IL-1-induced glycosaminoglycan production by cultured human orbital fibroblasts^64^. IL1RA could also block the induction of prostaglandin endoperoxidase H synthase-2 by leukoregulin^56^. A study had shown that upon cytokines exposure, markedly lower level of IL1RA expression was found in cultured orbital fibroblasts of GO patients as compared to the normal orbital fibroblasts^65^. Although *IL1RA* A2/non-A2 was not significant in our meta-analysis, another *IL1RA* SNP (c.11100C>T) had shown positive association in Iranians^21^. Follow-up studies on the *IL1RA* polymorphisms are needed to confirm the role of *IL1RA* in GO in specific population such as Iranians.

IL4 is a potent Th2 cytokine which stimulates proliferation of IgE- and IgG- secreting B cells and the expression of HLA class II antigens via STAT6^66^ against Th1 inflammatory response^30^. It has also been detected in orbital fat tissues of GO patients^12^. The promoter SNP rs2070874, which has transcriptional activity^30^,^67^, did not show a significant association with GO in our meta-analysis. However, significant associations of other *IL4* SNPs, including c.-1098T>G and c.-33C>T, with GO have been reported in Iranians^31^. Therefore, these two *IL4* polymorphisms should be tested in future studies. *IL13* and *IL4* have similar biological functions^68^. *IL13* is an anti-inflammatory cytokine that regulates IgE synthesis^68^,^69^ and the maturation of B cells^30^. However, there was no significant association detected in Chinese (rs1800925)^30^, Japanese (rs1800925 and c.-2044G>A)^37^ and Caucasians (rs1800925 and c.-2044G>A)^36^. Consistently, we did not detect associations of these two *IL13* SNPs with GO.

The *IL6* SNP rs1800795 is associated with multiple autoimmune diseases, including systemic-onset juvenile chronic arthritis^70^, type I diabetes mellitus^71^, rheumatoid arthritis^72^ and Sjogren’s syndrome^73^. When compared with healthy controls, serum IL6 levels were significantly higher in GD and GO patients, especially in active GO patients^74^. However, only one SNP rs1800795 in *IL6* was eligible for the meta-analysis, and it showed a lack of significant association.

This meta-analysis also reveals several limitations in the existing genetic studies of GO. First, the small number of published genetic studies on IL-related genes in GO limited the power of determining the associations, especially among different ethnic groups. Second, GO may not develop concurrently with GD. Classifying GO based on a cross-sectional assessment of observer-dependent signs and subject-dependent symptoms may therefore introduce bias. Third, as the pathogenesis of GO and GD is multifactorial, it would be more informative to test genetic, environmental (e.g. smoking), hormonal (e.g. fluctuation of thyroid function) and antigenic (thyroid related autoantibodies and use of radioactive iodine) factors, and their interactions in the study population. However, few genetic studies on IL-related genes and GO provided such information.

In conclusion, in this systematic review and meta-analysis of the association of IL-related genes with GO, we identified *IL1A* rs1800587 as the only SNP that is potentially associated with GO. Since the overall number of studies is small, further studies with larger sample sizes are needed to confirm *IL1A* rs1800587 as a genetic biomarker for GO, and also verify the roles of other IL-related genes in the disease.

## Methods

### Searching Strategy

We performed the literature search in the EMBASE and MEDLINE databases via the Ovid platform using structured search strategies. We identified citations recorded during the period starting from May 1, 1989 to February 25, 2015. Boolean logic and search terms with controlled vocabularies (i.e. Medical Subject Heading terms) were used: (Graves’ ophthalmopathy OR thyroid associated ophthalmopathy) AND interleukins (Supplementary Table 5). Moreover, we manually scanned the reference lists of the identified articles, reviews, and meta-analyses to include all potentially relevant articles. No language filters were applied in the literature search.

### Inclusion and Exclusion Criteria

A study was included if it fulfilled the following criteria: (1) original case-control study on the associations of IL-related genes polymorphisms with GO; (2) cases were patients with GO defined by clinical signs of GO or NOSPECS Class 2 or 3 and above; (3) controls were patients with Graves’ disease without GO (no clinical signs of GO or NOSPECS Class 0 or 1); (4) study subjects were unrelated individuals from clearly defined populations; (5) allele or genotype counts or frequencies in both case and control groups were provided (or existing data allow their calculation). Animal studies, case reports, reviews, abstracts, conference proceedings, editorials and studies with incomplete data were excluded.

### Literature Review and Data Extraction

Two investigators (W.K.H. and S.S.R.) screened and reviewed all studies independently. Disagreement was resolved by thorough discussion with a third investigator (L.J.C.) until consensus was reached. A customized data form was used to extract the data, which included the first author, year of publication, country of study, ethnicity, definition of cases and controls, sample size in case and control groups, gene and polymorphisms studied, allelic and genotypic counts, and result of the test for HWE in the control group. Two reviewers (W.K.H. and S.S.R.) extracted the data independently. Disagreement was resolved by consensus among the investigators. If the allele counts were not reported, we calculated them from the genotype data. If genotype counts were missing, we estimated the data using allele frequencies (if available) and sample sizes, assuming no deviation from HWE unless otherwise reported^75^. If there was no extractable genetic information in an eligible study, we communicated with the authors for the data. Allele counts of the eligible SNPs for meta-analysis were summarized in Supplementary Table 6.

### Statistical Analysis

Meta-analysis for each gene polymorphism was performed if it was reported in 2 or more studies. The genetic association was assessed using different genetic models, including allelic (A vs. a), dominant (AA+Aa vs. aa), recessive (aa vs. AA+Aa) and codominant (homozygous: AA vs. aa; heterozygous: AA vs. Aa) models. The strength of association was evaluated using the summary odds ratios and 95% confidence intervals of each gene polymorphism. Heterogeneity was tested by the Q-statistic and the *I*^*2*^ value^76^. The Q-statistic was considered significant when P < 0.10. The *I*^*2*^ values indicated no (0–24.9%), low (25–49.9%), moderate (50–74.9%) or high (75–100%) inter-study heterogeneity^76^,^77^. If the *P* value for the Q-statistic was <0.10 or the *I*^*2*^ value ≥ 50%, a random-effect model was used, otherwise a fixed-effect model was adopted^78^. In the assessment of data quality, we first examined the HWE in the control groups. If HWE was not reported, we tested it using the control group data with the Chi-square test. Also, we adopted the Newcastle Ottawa Scale (accessed via http://www.ohri.ca/programs/clinical_epidemiology/oxford.asp) to evaluate the quality of case-control studies (**Appendix 1**). A star was given to each study if one requirement in the NOS from 3 dimensions (selection, comparability and exposure) was met. The maximum number of stars that can be assigned to one study was 9. A study obtaining ≤6 stars was considered as of high risk in introducing bias^79^. We then conducted a sensitivity analysis to confirm the associations by sequentially omitting each of the studies one at a time, studies deviated from HWE, or studies of suboptimal quality^80^. Furthermore, the Funnel plots and Egger’s test were performed to assess potential biases (e.g. publication bias)^81^,^82^. The presence of bias was suggested when the P value of the Egger’s test was <0.05. All statistical analyses were performed using the R software for statistical computing (v3.0.0, http://cran.r-project.org/). Of note, since we tested the genetic association using 5 genetic models, the risk of type I error might be increased; therefore, we corrected the P values for association using the Bonferroni method. Thus, a P value of less than 0.010 (0.05/5) was considered statistically significant, where 5 is the number of genetic models being tested.

## Additional Information

**How to cite this article**: Wong, K. H. *et al.* Genetic Associations of Interleukin-related Genes with Graves’ Ophthalmopathy: a Systematic Review and Meta-analysis. *Sci. Rep.* **5**, 16672; doi: 10.1038/srep16672 (2015).

## Supplementary Material

Supplementary Information

## Figures and Tables

**Table 1:** Characteristics of studies included in the meta-analysis.

No.	Study (year)	Country	Ethnicity	Definition		Sample size(Cases/controls)	Gene	Test for HWE	Adjusted factor
				Cases	Controls				
1	Cuddihy RM (1996)	USA	Caucasians	GO	GD without GO	98/28	*IL1RA*	Not reported	Not reported
2	Muhlberg T (1998)	Germany	Caucasians	GO	GD without GO	44/100	*IL1RA*	Not reported	Not reported
3	Bednarczuk T (2003)	Poland	Caucasians	GO	GD without GO	93/168	*IL13*	Not reported	Not reported
4	Bednarczuk T (2004)	Poland	Caucasians	GO	GD without GO	108/171	*IL6*	Not reported	Age, sex, smoking status
5	Yang Y (2005)	China	Chinese	GO	GD without GO	98/89	*IL4* and *IL13*	In HWE	Age of onset
6	Hiromatsu Y (2005)	Japan	Japanese	GO	GD without GO	98/212	*IL13*	In HWE	Smoking status
7	Hiromatsu Y (2006)	Japan	Japanese	GO	GD without GO	103/226	*IL12B*	In HWE	Not reported
8	Huber AK (2008)	USA	Caucasians	GO	GD without GO	103/111	*IL23R*	Not reported	Not reported
9	Ban Y (2009)	Japan	Japanese	GO	GD without GO	100/190	*IL 23R*	Not reported	Not reported
10	Lacka K (2009)	Poland	Caucasians	GO^a^	GD with GO^b^	75/42	*IL1B*	In HWE	Not reported
11	Khalilzadeh O (2009)	Iran	Iranian	GO	GD without GO	50/57	*IL1A, IL1B, IL1R,* and *IL1RA*	In HWE	Family history of GD, age of onset, duration of GD
12	Anvari M (2010)	Iran	Iranian	GO	GD without GO	50/57	*IL2, IL6,* and *IL12B*	In HWE	Not reported
13	Liu N (2010)	China	Chinese	GO	GD without GO^c^	190/569	*IL1A* and *IL1B*	In HWE	Not reported
14	Zhu W (2010)	China	Chinese	GO	GD without GO^c^	190/561	*IL3, IL4, IL5, IL9,* and *IL13*	In HWE	Not reported
15	Liu YH (2010)	China	Chinese	GO	GD without GO	200/271	*IL1B*	Not reported	Age, sex, smoking status
16	Khalilzadeh O (2010)	Iran	Iranian	GO	GD without GO	50/57	*IL4* and *IL10*	In HWE	Not reported

GD: Grave’s Disease; GO: Grave’s Ophthalmopathy; HWE: Hardy Weinberg equilibrium; IL1A: Interleukin 1 Alpha; IL1B: Interleukin 1 Beta; IL1R: Interleukin 1 Receptor; IL1RA: Interleukin 1 Receptor Antagonist; IL12B: Interleukin 12 Beta; IL23R: Interleukin 23 Receptor; No.: number.

^a^GD with GO of NOSPECS class III or greater from the onset.

^b^GD with GO developed during 6 months to 7 years from GD onset.

^c^GD without GO of NOSPECS 0 and 1.

^d^NOSPECS classification system for the severity of GO (Supplementary Table 1).

**Table 2:** Meta-analysis of interleukin-related gene polymorphisms in Graves’ ophthalmopathy.

Gene	Polymorphism	No. ofcohorts	Ethnicity	Geneticmodel	Total allele orgenotype counts		FEM or REM^a^		Heterogeneity		Egger’stest (P)
					Case	Control	OR (95% CI)	P	P (Q)	I^2^ (%)	
*IL1A*	rs1800587	2	Chinese, Iranian	T vs. C	480	1252	1.62 (1.00–2.62)	**0.050**	0.14	53.7	n.a.
				TT vs. TC + CC	240	626	2.39 (1.07–5.37)	**0.039**	0.25	23.6	n.a.
				TT + TC vs. CC	240	626	1.80 (0.86–3.78)	0.12	0.15	51.7	n.a.
				TT vs. CC	172	500	2.66 (0.41–17.22)	0.31	0.13	57.0	n.a.
				TC vs. CC	221	613	1.52 (1.04–2.22)	**0.034**	0.37	0.0	n.a.
*IL1B*	rs1143634	3	Chinese, Caucasians, Iranian,	T vs. C	650	736	0.93 (0.62–1.40)	0.72	0.85	0.0	0.61
				TT vs. TC + CC	325	368	1.02 (0.62–1.67)	0.94	0.73	0.0	0.75
				TT + TC vs. CC	325	368	0.37 (0.10–1.32)	0.13	0.85	0.0	0.82
				TT vs. CC	89	63	0.38 (0.10–1.40)	0.15	0.92	0.0	0.85
				TC vs. CC	247	308	0.33 (0.09–1.22)	0.10	0.72	0.0	0.84
*IL1B*	rs16944	4	Chinese, Caucasians, Iranian	C vs. T	1030	1880	1.09 (0.93–1.28)	0.29	0.69	0.0	0.89
				CC vs. CT + TT	515	940	1.11 (0.87–1.40)	0.41	0.97	0.0	0.68
				CC + CT vs. TT	515	940	1.13 (0.83–1.54)	0.45	0.15	38.9	0.95
				CC vs. TT	261	455	1.18 (0.83–1.67)	0.36	0.31	7.2	0.85
				CT vs. TT	333	650	1.10 (0.79–1.52)	0.58	0.11	46.2	0.90
*IL1RA*	A2/non-A2^b^	2	Caucasians	A2 vs. Non-A2	284	256	1.45 (0.89–2.35)	0.13	0.68	0.0	n.a.
*IL4*	rs2070874	3	Chinese, Iranian	T vs. C	333	692	0.93 (0.74–1.17)	0.52	0.30	22.3	0.37
				TT vs. TC + CC	333	692	0.88 (0.66–1.18)	0.39	0.44	7.5	0.26
				TT + TC vs. CC	333	692	0.95 (0.54–1.65)	0.84	0.16	0.0	0.060
				TT vs. CC	212	457	1.16 (0.54–2.47)	0.71	0.18	41.3	0.1
				TC vs. CC	154	284	0.97 (0.55–1.72)	0.92	0.15	0.0	0.050
*IL6*	rs1800795	2	Caucasians, Iranian	C vs. G	316	456	1.29 (0.66–2.54)	0.45	0.040	77.3	n.a.
				CC vs. CG + GG	158	228	1.48 (0.65–3.37)	0.36	0.12	58.3	n.a.
				CC + CG vs. GG	158	228	1.53 (0.41–5.78)	0.53	0.040	76.5	n.a.
				CC vs. GG	78	107	2.04 (0.36–11.64)	0.42	0.020	80.9	n.a.
				CG vs. GG	119	184	1.34 (0.43–4.20)	0.62	0.080	67.2	n.a.
*IL12B*	rs3212227	2	Japanese, Iranian	A vs. C	306	566	0.60 (0.26–1.39)	0.23	0.010	85.4	n.a.
				AA vs. AC + CC	153	283	0.46 (0.13–1.66)	0.24	0.020	82.5	n.a.
				AA + AC vs. CC	153	283	0.57 (0.20–1.59)	0.28	0.070	68.8	n.a.
				AA vs. CC	74	147	0.35 (0.06–2.04)	0.24	0.010	84.2	n.a.
				AC vs. CC	123	202	0.81 (0.49–1.32)	0.39	0.30	7.0	n.a.
*IL13*	rs1800925	3	Chinese, Japanese, Caucasians	C vs. T	566	904	1.08 (0.82–1.41)	0.59	0.27	29.5	0.17
				CC vs. CT + TT	283	452	1.14 (0.83–1.58)	0.42	0.51	0.0	0.70
				CC + CT vs. TT	283	452	0.88 (0.45–1.73)	0.71	0.27	30.2	0.12
				CC vs. TT	200	301	0.92 (0.46–1.84)	0.82	0.26	31.5	0.11
				CT vs. TT	99	175	0.84 (0.41–1.71)	0.63	0.36	12.2	0.19
*IL13*	c.-2044G>A	2	Japanese, Caucasians	G vs. A	382	760	1.02 (0.77–1.36)	0.87	0.31	2.3	n.a.
				GG vs. GA + AA	191	380	0.99 (0.70–1.41)	0.97	0.56	0.0	n.a.
				GG + GA vs. AA	191	380	1.14 (0.55–2.34)	0.73	0.18	45.5	n.a.
				GG vs. AA	117	237	1.12 (0.54–2.35)	0.76	0.17	46.4	n.a.
				GA vs. AA	86	171	1.18 (0.55–2.52)	0.67	0.22	34.9	n.a.
*IL23R*	rs10889677	2	Caucasians, Japanese	C vs. A	400	604	1.15 (0.46–2.86)	0.76	0.0018	89.7	n.a.
				CC vs. CA + AA	200	302	1.40 (0.46–4.21)	0.55	0.040	77.1	n.a.
				CC + CA vs. AA	200	302	0.93 (0.37–2.32)	0.87	0.12	58.5	n.a.
				CC vs. AA	132	163	1.20 (0.31–4.66)	0.80	0.070	70.1	n.a.
				CA vs. AA	129	238	0.72 (0.44–1.15)	0.17	0.44	0.0	n.a.
*IL23R*	rs2201841	2	Caucasians, Japanese	A vs. G	402	606	1.20 (0.55–2.63)	0.65	0.010	86.3	n.a.
				AA vs. AG + GG	201	303	1.60 (0.70–3.66)	0.26	0.10	64.0	n.a.
				AA + AG vs. GG	201	303	0.80 (0.51–1.25)	0.33	0.23	30.2	n.a.
				AA vs. GG	133	163	1.17 (0.58–2.36)	0.65	0.19	41.5	n.a.
				AG vs. GG	127	238	0.72 (0.45–1.16)	0.18	0.62	0.0	n.a.
*IL23R*	rs7530511	2	Caucasians, Japanese	C vs. T	396	580	0.68 (0.39–1.17)	0.16	0.41	0.0	n.a.
				CC vs. CT + TT	198	290	0.64 (0.35–1.18)	0.15	0.43	0.0	n.a.
				CC + CT vs. TT	198	290	0.64 (0.12–3.32)	0.60	0.90	0.0	n.a.
				CC vs. TT	168	268	0.59 (0.11–3.09)	0.53	0.93	0.0	n.a.
				CT vs. TT	33	24	0.98 (0.17–5.50)	0.98	0.80	0.0	n.a.

CI: Confidence interval; FEM: fixed effects model; GD: Grave’s Disease; GO: Graves’ Ophthalmopathy; IL: Interleukin; *IL1A*: Interleukin 1 Alpha; *IL1B*: Interleukin 1 Beta; *IL1RA*: Interleukin 1 Receptor Antagonist; *IL12B*: Interleukin 12 Beta; *IL23R*: Interleukin 23 Receptor; No.: number; OR: Odds ratio; REM: random effects model.

^a^If the P value for Q-statistic was <0.10 or the I^2^ value ≥ 50%, a random-effects model was used, otherwise a fixed-effects model was adopted.

^b^A2 = 2 repeats of a 86-bp segment; non-A2 = other number of repeats of a 86-bp segment.
