# Supplementary material for: Genetic Associations of Interleukin-related Genes with Graves’ Ophthalmopathy: a Systematic Review and Meta-analysis
Source: Sci Rep. 2015 Nov 18;5:16672. doi: 10.1038/srep16672 (PMC4649612; doi:10.1038/srep16672)
Supplement: Supplementary Information [file srep16672-s1.pdf]

Genetic Associations of Interleukin-related Genes with Graves' Ophthalmopathy: a Systematic Review and Meta-analysis

Kah Hie Wong[1*], Shi Song Rong[1*], Kelvin K.L. Chong[1,2], Alvin L. Young[1,2], Chi Pui Pang[1], Li Jia Chen[1,2]

[1] Department of Ophthalmology and Visual Sciences, The Chinese University of Hong Kong, Hong Kong, China;
[2] Department of Ophthalmology and Visual Sciences, Prince of Wales Hospital, Hong Kong, China.

* contributed equally to the study

Number of supplementary information: 6 supplementary tables and 1 appendix

Correspondence:
Dr Li Jia Chen
Department of Ophthalmology and Visual Sciences, The Chinese University of Hong Kong, Hong Kong Eye Hospital, 147K, Argyle Street, Kowloon, Hong Kong.
Tel: +852 39435810
Fax: +852 27159490
E-mail: lijia_chen@cuhk.edu.hk

Supplementary Table 1. NOSPECS classification

N	No signs or symptoms
O	Only signs
S	Soft tissue symptoms and signs
P	Proptosis
E	Extraocular muscle involvement
C	Corneal involvement
S	Sight loss (optic nerve involvement)

Supplementary Table 2. Subgroup analysis in Chinese population

Gene	Polymorphism	No. of cohorts	Genetic model	Total allele or genotype counts		Fixed Effects Model		Random Effects Model		Heterogeneity	
				Cases	Controls	OR (95% CI)	P	OR (95% CI)	P	P for Q	I^2 (%)
IL1B	rs16944	2	C vs. T	780	1,682	1.08 (0.91-1.29)	0.38	1.09 (0.91-1.29)	0.38	0.49	0.0
		2	CC vs. CT + TT	390	841	1.08 (0.83-1.41)	0.56	1.08 (0.83-1.41)	0.56	0.92	0.0
		2	CC + CT vs. TT	390	841	1.13 (0.81-1.57)	0.48	1.12 (0.66-1.89)	0.67	0.12	59.3
		2	CC vs. TT	188	404	1.17 (0.80-1.70)	0.42	1.17 (0.77-1.78)	0.47	0.26	20.3
		2	CT vs. TT	265	588	1.11 (0.78-1.57)	0.58	1.09 (0.61-1.97)	0.76	0.093	64.5
IL4	rs2070874	2	T vs. C	566	1,270	0.93 (0.72-1.19)	0.57	1.01 (0.64-1.59)	0.98	0.12	58.7
		2	TT vs. TC + CC	283	635	0.86 (0.64-1.16)	0.34	0.87 (0.63-1.19)	0.38	0.30	5.8
		2	TT + TC vs. CC	283	635	1.18 (0.52-2.68)	0.69	1.95 (0.26-14.34)	0.51	0.077	68.1
		2	TT vs. CC	184	430	1.10 (0.48-2.52)	0.82	1.86 (0.24-14.77)	0.56	0.069	69.8
		2	TC vs. CC	107	229	1.35 (0.58-3.14)	0.49	2.09 (0.32-13.67)	0.44	0.10	62.9

CI=Confidence interval; IL1B=Interleukin 1 Beta; IL4=Interleukin 4; No.=number; OR=Odds ratio.

Supplementary Table 3. Sensitivity analysis by excluding Lacka's study

Gene	Polymorphism	No. of cohorts	Ethnic group	Genetic model	Total allele or genotype counts		Fixed Effects Model		Random Effects Model		Heterogeneity	
					Cases	Controls	OR (95% CI)	P	OR (95% CI)	P	P for Q	I^2 (%)
IL1B	rs16944	3[a]	Chinese, Iranian	C vs. T	880	1,796	1.11 (0.94-1.31)	0.24	1.11 (0.94-1.31)	0.24	0.56	0.0
				CC vs. CT + TT	440	898	1.10 (0.86-1.42)	0.45	1.10 (0.86-1.42)	0.45	0.90	0.0
				CC + CT vs. TT	440	898	1.19 (0.86-1.64)	0.29	1.24 (0.76-2.04)	0.39	0.14	48.0
				CC vs. TT	209	430	1.24 (0.86-1.78)	0.24	1.26 (0.83-1.89)	0.28	0.28	16.1
				CT vs. TT	298	629	1.17 (0.83-1.63)	0.37	1.22 (0.71-2.08)	0.47	0.13	51.0
IL1B	rs1143634	2[a]	Chinese, Iranian	T vs. C	500	652	1.11 (0.66-1.88)	0.69	1.11 (0.66-1.88)	0.69	0.59	0.0
				TT vs. TC + CC	250	326	2.11 (0.43-10.38)	0.36	2.11 (0.43-10.38)	0.36	0.81	0.0
				TT + TC vs. CC	250	326	1.16 (0.61-2.18)	0.66	1.16 (0.61-2.18)	0.66	0.96	0.0
				TT vs. CC	31	36	2.21 (0.43-11.35)	0.34	2.21 (0.43-11.35)	0.34	0.85	0.0
				TC vs. CC	246	324	1.09 (0.57-2.09)	0.79	1.09 (0.57-2.09)	0.79	0.86	0.0

CI=Confidence interval; IL1B=Interleukin 1 Beta; OR=Odds ratio; No.=number.

[a] Control subjects were defined as GD without GO; Lacka's study was excluded [1].

Reference

1 Lacka, K. *et al.* Interleukin-1beta gene (IL-1beta) polymorphisms (SNP -511 and SNP +3953) in thyroid-associated ophthalmopathy (TAO) among the Polish population. *Curr Eye Res* **34**, 215-220, (2009).

Supplementary Table 4. Quality assessments of included case-control studies using the Newcastle Ottawa Scale

No.	Study (year)	Selection				Comparability		Exposure			Total no. of stars
		1	2	3	4	1(a)	1(b)	1(a)	2	3	
1	Cuddihy RM (1996)	*	*	-	*	*	*	*	*	*	8
2	Muhlberg T (1998)	*	*	-	*	*	*	*	*	*	8
3	Bednarczuk T (2003)	*	*	-	*	*	*	*	*	*	8
4	Bednarczuk T (2004)	*	*	-	*	*	*	*	*	*	8
5	Yang Y (2005)	*	-	-	*	*	*	*	*	*	7
6	Hiromatsu Y (2005)	*	*	-	*	*	*	*	*	*	8
7	Hiromatsu Y (2006)	*	*	-	*	*	*	*	*	*	8
8	Huber AK (2008)	-	*	-	*	*	*	*	*	*	7
9	Ban Y (2009)	-	*	-	*	*	*	*	*	*	7
10	Lacka K (2009)	*	*	-	*	*	-	*	*	*	7
11	Khalilzadeh O (2009)	*	*	-	*	*	*	*	*	*	8
12	Anvari M (2010)	*	*	-	*	*	*	*	*	*	8
13	Liu N (2010)	*	*	*	*	*	-	*	*	*	8
14	Zhu W (2010)	*	*	-	*	*	-	*	*	*	7
15	Liu YH (2010)	*	*	-	*	*	-	*	*	*	7
16	Khalilzadeh O (2010)	*	*	-	*	*	*	*	*	*	8

"-" means no star was assigned.

Supplementary Table 5. Searching strategies used in Ovid platform

1	((thyroid associated or thyroid-associated) and (orbitopathy or orbitopathies or ophthalmopathy or ophthalmopathies)).tw.
2	(endocrine and (orbitopathy or orbitopathies or ophthalmopathy or ophthalmopathies)).tw.
3	ophthalmic Graves disease.tw.
4	(thyroid and (orbitopathy or orbitopathies or ophthalmopathy or ophthalmopathies)).tw.
5	(Graves adj1 (orbitopathy or orbitopathies or ophthalmopathy or ophthalmopathies)).tw.
6	(dysthyroid and (orbitopathy or orbitopathies or ophthalmopathy or ophthalmopathies)).tw.
7	exp endocrine ophthalmopathy/
8	1 or 2 or 3 or 4 or 5 or 6 or 7
9	exp Interleukins/
10	(interleukin or interleukins).mp.
11	9 or 10
12	8 and 11

Supplementary Table 6. Allele information of polymorphisms eligible for meta-analysis

No.	Author (year)	Sample size		Gene	Polymorphism	Definition of allele		Allele count (A1/A2)	
		Cases	Controls			A1	A2	Cases	Controls
1	Cuddihy, R. M. (1996)	98	28	*IL1RA*	A2/non-A2*	A2	Non-A2	47 / 149	9 / 47
2	Muhlberg, T. (1998)	44	100	*IL1RA*	A2/non-A2*	A2	Non-A2	20 / 68	36 / 164
3	Bednarczuk, T. (2003)	93	168	*IL13*	rs1800925	T	C	61 / 125	99 / 237
		93	168	*IL13*	c.-2044G>A	A	G	50 / 136	82 / 254
4	Bednarczuk, T. (2004)	108	171	*IL6*	c.-174G>C	C	G	95 / 121	155 / 187
5	Hiromatsu, Y. (2005)	98	212	*IL13*	rs1800925	C	T	170 / 26	351 / 73
		98	212	*IL13*	c.-2044G>A	G	A	148 / 48	307 / 117
6	Yang, Y. (2005)	92	72	*IL13*	rs1800925	C	T	156 / 28	117 / 27
		98	89	*IL4*	rs2070874	T	C	166 / 30	143 / 35
7	Hiromatsu, Y. (2006)	103	226	*IL12B*	c.-1188A>C	A	C	95 / 111	221 / 231
8	Huber, A. K. (2008)	103	111	*IL23R*	rs10889677	C	A	162 / 44	148 / 74
		103	111	*IL23R*	rs11209026	G	A	191 / 11	184 / 14
		103	111	*IL23R*	rs2201841	A	G	164 / 44	151 / 73
		103	111	*IL23R*	rs7530511	C	T	168 / 34	173 / 25
9	Ban, Y. (2009)	100	190	*IL23R*	rs10889677	A	C	146 / 48	263 / 119
		100	190	*IL23R*	rs2201841	G	A	142 / 52	263 / 119
		100	190	*IL23R*	rs7530511	C	T	192 / 2	381 / 1
10	Lacka, K. (2009)	75	42	*IL1B*	rs16944	C	T	103 / 47	59 / 25
11	Khalilzadeh, O. (2009)	50	57	*IL1A*	rs1800587	T	C	62 / 38	49 / 65
		50	57	*IL1B*	rs1143634	T	C	32 / 68	31 / 79
		50	57	*IL1B*	rs16944	C	T	63 / 37	63 / 51
12	Anvari, M. (2010)	50	57	*IL12B*	c.-1188A>C	A	C	44 / 56	77 / 37
		50	57	*IL6*	c.-174C>G	G	C	37 / 63	60 / 54
13	Liu, N. (2010)	190	570	*IL1A*	rs1800587	C	T	336 / 44	1,035 / 103

No.	Author (year)	Sample size		Gene	Polymorphism	Definition of allele		Allele count (A1/A2)	
		Cases	Controls			A1	A2	Cases	Controls
		190	570	*IL1B*	rs16944	C	T	223 / 157	632 / 508
14	Zhu, W. (2010)	190	561	*IL4*	rs2070874	T	C	285 / 85	874 / 218
15	Liu, Y.H. (2010)	200	271	*IL1B*	rs1143634	T	C	5 / 395	8 / 534
		200	271	*IL1B*	rs16944	C	T	229 / 171	309 / 233
16	Khalilzadeh, O. (2010)	50	57	*IL4*	rs2070874	C	T	72 / 28	80 / 34

* A2=2 repeats of a 86-bp segment; non-A2=other number of repeats of a 86-bp segment

Appendix 1

NEWCASTLE - OTTAWA QUALITY ASSESSMENT SCALE

CASE CONTROL STUDIES

(accessed via http://www.ohri.ca/programs/clinical_epidemiology/oxford.asp)

Note: A study can be awarded a maximum of one star for each numbered item within the Selection and Exposure categories. A maximum of two stars can be given for Comparability.

Selection

1) Is the case definition adequate?

a) yes, with independent validation

b) yes, e.g. record linkage or based on self reports

c) no description

2) Representativeness of the cases

a) consecutive or obviously representative series of cases

b) potential for selection biases or not stated

3) Selection of Controls

a) community controls

b) hospital controls

c) no description

4) Definition of Controls

a) no history of disease (endpoint)

b) no description of source

Comparability

1) Comparability of cases and controls on the basis of the design or analysis

a) study controls for ________________ (Select the most important factor: ethnicity)

b) study controls for any additional factor (This criteria could be modified to indicate specific control for a second important factor.)

Exposure

1) Ascertainment of exposure (genotyping)

a) secure record (e.g. surgical records)

b) structured interview where blind to case/control status

c) interview not blinded to case/control status

d) written self report or medical record only

e) no description

2) Same method of ascertainment for cases and controls

a) yes

b) no

3) Non-Response rate (genotype call rate)

a) same rate for both groups

b) non respondents described

c) rate different and no designation
